# Comparative Analysis of the Evolutionary Dynamics of Seasonal Influenza Viruses in Madagascar Before and Since the Pandemic Period of COVID‐19

**DOI:** 10.1111/irv.70110

**Published:** 2025-05-07

**Authors:** Norosoa Harline Razanajatovo, Tsiry Hasina Randriambolamanantsoa, Laurence Randrianasolo, Joelinotahiana Hasina Rabarison, Sitraka Ulrich Raveloson, Baholy Barasaona, Nirina Nantenaina Ranoelison, Arvé Ratsimbazafy, Helisoa Razafimanjato, Fara Adèle Raveloharivony, Aina Harimanana, Rindra Randremanana, Miamina Fidy Ankasitrahana, Antso Hasina Raherinandrasana, Jean‐Michel Heraud, Philippe Dussart, Vincent Lacoste

**Affiliations:** ^1^ National Influenza Center, Virology Unit, Institut Pasteur de Madagascar Antananarivo Madagascar; ^2^ Epidemiology and Clinical Research Unit, Institut Pasteur de Madagascar Antananarivo Madagascar; ^3^ Direction de la Veille Sanitaire, de la Surveillance Epidémiologique et Ripostes, Ministry of Public Health Antananarivo Madagascar

**Keywords:** COVID‐19, evolution, genetics, influenza, Madagascar

## Abstract

Madagascar has maintained an influenza surveillance program for decades. Following the emergence of SARS‐CoV‐2 in 2020, the country implemented strict nonpharmaceutical interventions (NPIs) that disrupted influenza circulation. We studied the evolutionary dynamics of influenza viruses in Madagascar over a 5‐year period, spanning both the pre‐pandemic and COVID‐19 pandemic periods: 2019–2023. We showed that global genetic evolution profiles for A(H1N1)pdm09, A(H3N2), and B/Victoria viruses occurred from the pre‐pandemic to the pandemic period of COVID‐19 in Madagascar. In addition, we observed distinct patterns of viral re‐emergence following the relaxation of COVID‐19 containment measures. This study underscores the importance of sustaining continuous surveillance of influenza virus circulation to monitor the emergence of new variants and identify clade‐specific isolates. In addition, these results suggest that targeted NPIs could complement vaccination strategies in reducing influenza transmission and should be integrated into a comprehensive approach for effective influenza control.

Influenza remains a significant global health threat particularly for vulnerable populations such as the elderly and individuals with underlying health conditions that are at higher risk of developing serious infections. Influenza viruses are known to cause repetitive outbreaks due to their rapid antigenic changes that lead to viral evasion of host immunity. Annually, seasonal influenza causes approximately 39 million cases of acute respiratory illness and 58,200 deaths worldwide [1]. Moreover, influenza pandemics can arise when novel strains appear from animals or as a result of genetic reassortment. The 1918–1919 influenza A(H1N1) pandemic resulted in devastating human losses with an estimate of 50–80 million deaths globally [2]. The influenza A(H1N1)pdm09 resulted in less mortality, but still substantial, with an estimated 123,000 to 203,000 deaths during its first circulation [3].

To mitigate the impact of influenza, it is essential to constantly monitor circulating strains to understand how the virus evolves and to rapidly detect emerging strains that could pose a risk to public health. Madagascar has maintained an influenza surveillance program for almost five decades. The country is part of the Global Influenza Surveillance and Response System (GISRS) and shares influenza strains with WHO Collaborating Centers for antigenic and genetic characterization, as well as vaccine strain selection.

Influenza activity in Madagascar exhibits an irregular pattern, typically characterized by multiple peaks during both the hot, rainy season and the cooler, dry season [4, 5]. Following the detection of the first cases of SARS‐CoV‐2 in the country in March 2020, Malagasy authorities implemented stringent non‐pharmaceutical interventions (NPIs) such as lockdowns, travel restrictions, social distancing, mask wearing, hand hygiene practices, and school closures, to limit the spread of the virus (Figure 1). These measures were effective in curbing the transmission of COVID‐19. They have also disrupted the circulation of seasonal respiratory viruses, with no cases of influenza detected between March 2020 and July 2021. Influenza (B/Victoria) was detected again at the end of July 2021, causing a major epidemic from November 2021, followed by epidemics of varying intensity of influenza A viruses (Figure 1) [6]. It remained to be determined whether these outbreaks were due to newly introduced or previously circulating strains.

**FIGURE 1:**
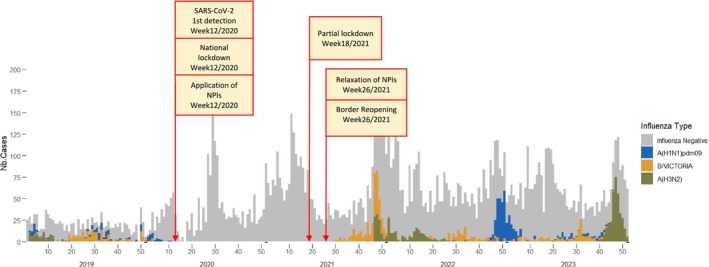
Temporal distribution of influenza viruses (type and subtypes), Madagascar, January 2019 to December 2023. Influenza negative samples are indicated in grey. The weeks of implementation of mitigation measures to fight against the propagation of SARS‐CoV‐2 and of their relaxation are indicated in boxes.

This report presents the results of maximum likelihood (ML) phylogenetic analyses conducted on influenza hemagglutinin (HA) gene sequences to characterize the genetic profile of Malagasy strains. The analysis covers a five‐year period, from January 2019 to December 2023, spanning two distinct periods: the COVID‐19 pre‐pandemic period (January 2019 to February 2020) and the COVID‐19 pandemic period (July 2021 to December 2023). During this timeframe, our surveillance efforts identified a total of 1958 individuals with influenza. To characterize the circulating strains, we sent primary samples from 602 individuals to the WHO collaborative centers in London and Atlanta for sequencing. The samples selected were representative of different age groups, geographic regions, and periods of seasonal epidemics. The sequencing analysis yielded 382 sequences, including 152 influenza B/Victoria sequences, 128 influenza A(H3N2) sequences and 102 influenza A(H1N1)pdm09 sequences. Sequences were downloaded from GISAID, the global data science initiative [7].

ML phylogenetic analysis of the full‐length HA sequences of Malagasy influenza B/Victoria strains reveals an evolutionary transition from genetic clade V1A.1 to V1A.3a.2 (Figure 2). Strains detected from January to June 2019 belonged to genetic clade V1A.1, characterized by two amino acid deletions (at positions 162–163) in the HA1 subunit, while those detected from October 2019 to February 2020 were classified in the genetic clade V1A.3, displaying three amino acid deletions (at positions 162–164) in this subunit. Notably, the genetic clades V1A.1 and V1A.3 have been detected globally since 2016–2017 and 2018, respectively. In July 2021, influenza B/Victoria was the first subtype to reemerge in Madagascar, shortly after the relaxation of COVID‐19 containment measures. Interestingly, the genetic clade V1A.3 was identified again but with distinct mutations—E128K and T170I in HA1 and G528E in HA2—causing these strains to cluster separately from other contemporary V1A.3 strains. As these substitutions have not been reported elsewhere, this suggests that the virus likely circulated at low levels during the COVID‐19 containment period, accumulated mutations, and was re‐identified after the restrictions were lifted. However, we cannot exclude the possibility that the reported strains originate from an ancestral strain introduced from outside Madagascar after restrictions were lifted, the closest strain being B/Washington/110/2019. The new subclade 3a.2, which emerged from V1A.3, has been circulating in Madagascar since 2022.

**FIGURE 2:**
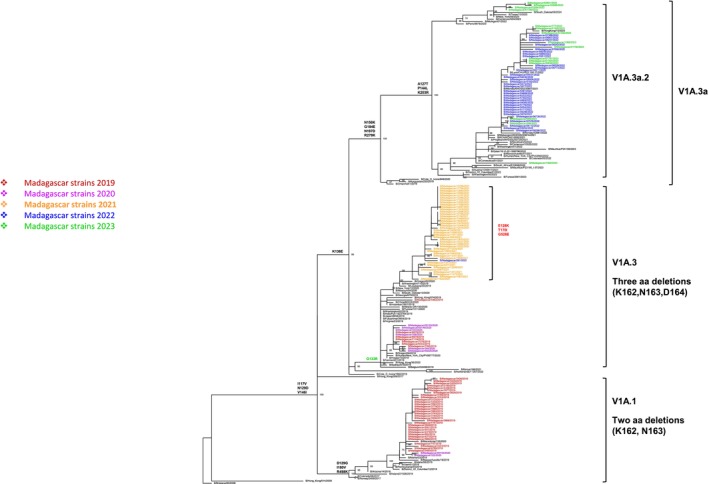
Phylogenetic tree of influenza B/Victoria based on HA complete coding sequences. Analyses were based on Maximum Likelihood estimation. The trees with the highest log‐likelihood are presented. The percentage of trees in which the associated taxa clustered together is indicated next to the branches (only values ≥ 70% are displayed). Substitutions indicating the temporal antigenic drift of strains are shown. Specific substitutions of Malagasy strains are highlighted in red.

Genetic changes have also occurred in A(H3N2) strains, which evolved from subclade 2 to 2a.3a.1, within the genetic clade 3C.2a1b (Figure 3). Phylogenetic analysis of complete HA sequences revealed that strains detected in 2019 belonged to subclade 2 and predominantly clustered with A/New Caledonia/19/2019, characterized by the K2E substitution. The A(H3N2) virus was identified again in November 2021 with subclade 2a.2c, which remained predominant until March 2022. These strains showed a close genetic relationship with Mayotte strains, sharing the D69N and R217K mutations. During the period from November 2021 to March 2022, two additional subclades, 2a.2a.2 and 1b, were also detected at lower frequencies. The concurrent circulation of multiple subclades in the country suggests multiple independent introductions of A(H3N2) viruses following the reopening of borders in August 2021. From April 2022, subclade 2a.2a was detected in Madagascar, which was subsequently replaced by 2a.2a.3a.1 in 2023.

**FIGURE 3:**
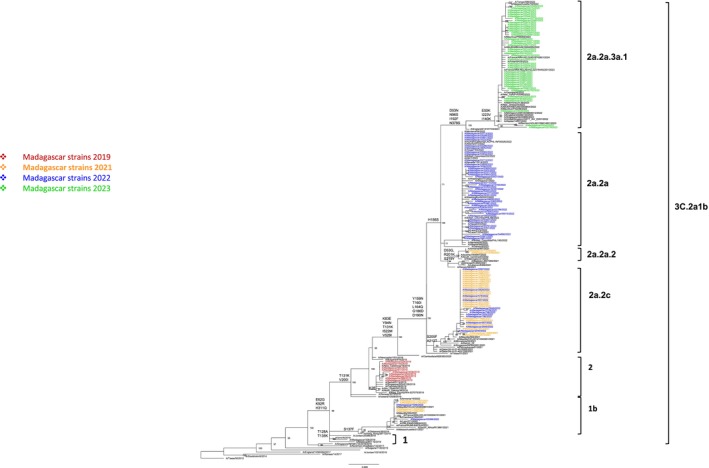
Phylogenetic tree of influenza A(H3N2) of the genetic group 3C.2a1b based on HA complete coding sequences. Analyses were based on Maximum Likelihood estimation. The trees with the highest log‐likelihood are presented. The percentage of trees in which the associated taxa clustered together is indicated next to the branches (only values ≥70% are displayed). Substitutions indicating the temporal antigenic drift of strains are shown.

Malagasy A(H1N1)pdm09 viruses evolved from subgroup 5a to 5a.2a within genotype 6B.1A over the study period (Figure 4). ML phylogenetic analysis revealed that strains isolated in between January 2019 and March 2020 fell into subgroup 5a, which was the predominant circulating genotype worldwide at that time. The genotype 5a is characterized by N129D, T185I and N260D substitutions in the HA1 subunit. The Malagasy strains, as well as the Qatari strain identified in 2019, also feature other substitutions, G172R and A302S, in HA1. Because of these additional features, these strains are grouped separately from the other members of this genotype. These distinctive characteristics suggest the circulation of specific strains in Madagascar prior to the COVID‐19 pandemic, which subsequently disappeared following the implementation of NPIs. The reemergence of A(H1N1)pdm09 was delayed compared to other influenza subtypes. The virus resurfaced in November 2022 with a new subgroup, 5a.2a, which had previously been reported across Australasia, Asia, Africa, the Middle East, and several American and European countries. One minor clade 5a.1 that includes sequences from La Reunion was also detected. The emergence of these new genotypes in Madagascar likely indicates novel introductions of strains into the country.

**FIGURE 4:**
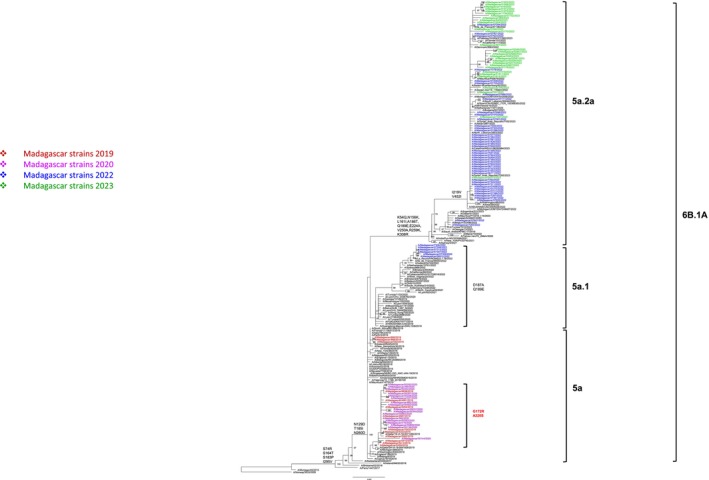
Phylogenetic tree of influenza A(H1N1)pdm09 of the genetic group 6B.1A based on HA complete coding sequences. Analyses were based on Maximum Likelihood estimation. The trees with the highest log‐likelihood are presented. The percentage of trees in which the associated taxa clustered together is indicated next to the branches (only values ≥ 70% are displayed). Substitutions indicating the temporal antigenic drift of strains are shown. Specific substitutions of Malagasy strains are highlighted in red.

Our results may be biased by limitations in surveillance capacity during the first months of the pandemic and subsequent waves, which led to challenges in specimen transportation from the country's sentinel sites to the National Influenza Centre. Consequently, a reduced number of samples from suspected influenza patients were collected in healthcare facilities outside the capital, Antananarivo. However, as illustrated in Figure 1, the overall number of samples tested remained relatively stable throughout the study period and, despite a possible geographical sampling bias, the surveillance system was robust enough to detect influenza circulation at community level.

In conclusion, this study underscores the crucial role of continuous influenza surveillance in monitoring the emergence of novel variants and characterizing isolates within specific clades. Tracking the genetic evolution of influenza strains remains essential for assessing vaccine effectiveness at both national and international levels. Our data reveal distinct patterns of reemergence of influenza viruses following COVID‐19 circulation and implementation of mitigation measures. Influenza B/Victoria genetic analyses are in favor of a circulation at low level during the implementation of NPIs. The virus then reemerged with accumulated mutations following the lifting of restrictions. Conversely, influenza A(H1N1)pdm09 and A(H3N2) viruses completely disappeared before appearing again with newly introduced strains. These results suggest that integrating specific NPIs, such as targeted mask use, could complement vaccination strategies in mitigating influenza transmission. Such a comprehensive approach, combining vaccination and strategic NPIs implementation, should be considered for effective influenza control.

## Author Contributions

**Norosoa Harline Razanajatovo:** conceptualization, methodology, software, funding acquisition, validation, formal analysis, data curation, writing – original draft. **Tsiry Hasina Randriambolamanantsoa:** methodology, software, validation, writing – review and editing. **Laurence Randrianasolo:** conceptualization, methodology, funding acquisition, software, formal analysis, investigation, data curation, writing – review and editing. **Joelinotahiana Hasina Rabarison:** methodology, software, formal analysis, investigation, data curation, writing – review and editing. **Sitraka Ulrich Raveloson:** methodology, investigation, data curation, writing – review and editing. **Baholy Barasaona:** investigation, data curation, writing – review and editing. **Nirina Nantenaina Ranoelison:** investigation, methodology, writing – review and editing, data curation. **Arvé Ratsimbazafy:** methodology, investigation, data curation, writing – review and editing. **Helisoa Razafimanjato:** methodology, validation, writing – review and editing. **Fara Adèle Raveloharivony:** methodology, formal analysis, investigation, writing – review and editing. **Aina Harimanana:** methodology, investigation, writing – review and editing. **Rindra Randremanana:** conceptualization, funding acquisition, project administration, supervision, writing – review and editing. **Miamina Fidy Ankasitrahana:** methodology, formal analysis, investigation, data curation, writing – review and editing. **Antso Hasina Raherinandrasana:** methodology, formal analysis, investigation, data curation, writing – review and editing. **Jean‐Michel Heraud:** conceptualization, funding acquisition, writing – review and editing, project administration, supervision. **Philippe Dussart:** supervision, conceptualization, funding acquisition, project administration, writing – review and editing. **Vincent Lacoste:** supervision, conceptualization, funding acquisition, project administration, validation, writing – review and editing.

## Ethics Statement

The data used were obtained from state‐wide surveillance of a notifiable disease and de‐identified and, thus, did not require ethical approval. Although the influenza surveillance in Madagascar is not considered research, our surveillance system and its objectives were submitted and approved by the National Ethics Committee of the Madagascar Ministry of Public Health (Authorizations N°096‐MSANP/ceRBM and 088‐MSANP/CE).

## Conflicts of Interest

The authors declare no conflicts of interest.

### Peer Review

The peer review history for this article is available at https://www.webofscience.com/api/gateway/wos/peer‐review/10.1111/irv.70110.

## Supporting information


**Data S1.** Supporting Information

**Data S2.** Metadata of Malagasy strains A(H1N1)pdm09 used in this study

**Data S3.** Metadata of Malagasy strains A(H3N2) used in this study

**Data S4.** Metadata of Malagasy strains B/Victoria used in this study

## Data Availability

The Malagasy sequences are available on GISAID. Metadata are provided in the Data S1 (for A(H1N1)pdm09), 2 (for A(H3N2)), and 3 (for B/Victoria).
