# Supplementary material for: Comparative Analysis of the Evolutionary Dynamics of Seasonal Influenza Viruses in Madagascar Before and Since the Pandemic Period of COVID‐19
Source: Influenza Other Respir Viruses. 2025 May 7;19(5):e70110. doi: 10.1111/irv.70110 (PMC12058301; doi:10.1111/irv.70110)
Supplement: Supplementary file 1 — Data S1. Supporting Information [file IRV-19-e70110-s003.docx]

**Laboratory protocols**

**Study sites**

Samples were collected as part of the influenza surveillance in Madagascar from January 1, 2019 to December 31, 2023. Influenza surveillance is based on two components: influenza-like illness (ILI) surveillance, which includes 21 sentinel sites, and severe acute respiratory infection (SARI) surveillance, which includes 5 hospitals. These sites are distributed across 15 of the 23 regions of the country.

**Study subjects**

All patients presenting with ILI or SARI or COVID-19 symptoms at consultation were enrolled. WHO case definitions for ILI and SARI were used for patient enrollment. ILI was defined as fever and cough not requiring hospitalization, with symptom onset within the last 10 days. SARI is defined as fever or history of fever and cough requiring hospitalization and duration of symptoms for less than 10 days. Suspicion of COVID-19 was based on clinical diagnosis. For each consenting patient, demographic, socio-economic, clinical, and epidemiological data were recorded in case report forms.

**Biological analysis**

Throat, nasal, or nasopharyngeal specimens were collected from each enrolled patient. Samples were place in Universal Transport Media (Copan, Italia) and shipped at 4°C two to three times a week to the National Influenza Center (NIC) laboratory at the Institut Pasteur de Madagascar, where they were immediately processed or stored at 4˚C until testing (test were performed within 48 hours after sampling). All specimens were tested for Influenza A and B viruses and SARS-CoV-2 using CDC influenza detection kits purchased from IRR (Influenza Reagent Resources). Specimens positive for influenza A or B viruses were then tested further to determine subtype (H1N1pdm09, H3) or sub-lineage (B/Victoria, B/Yamagata), respectively

**Samples preparation for Sequencing**

Positive samples were sent to the WHO Collaborating Center for Surveillance, Epidemiology and Control of Influenza, CDC in Atlanta, USA, or to the WHO Collaborating Centre for Reference and Research on Influenza, the Francis Crick Institute in London, England, for genomic sequencing. Samples were selected based on viral load (Ct value <30), age groups, geographic regions, and periods of seasonal epidemics.

**Phylogenetics Analysis**

All Malagasy Fasta and metadata files were downloaded from GISAID. Reference sequences were obtained from GISAID Audacity Instant, which selects sequences closely related to those from Madagascar, and from NCBI through BLAST alignment. Three ways have been used to add the reference sequences: from Audacity Instant of GISAID to select the closely related sequences of Madagascar, from NCBI by blast alignment, and sequences from countries that are geographically closed/connected to Madagascar.

Multiple sequence alignments were made using ClustalW embedded in Aliview software 1.21. Sequences were analyzed to identify substitutions, insertions or deletions in the studied strains. Phylogenetic analysis was performed using the maximum-likelihood method and phylogenetic trees were inferred in IQ-TREE version 2.0.7. All the model were inferred with ModelFinder and the best model was chosen using BIC (Bayesian Information Criterion). The statistical significance of the tree topology was assessed through 1,000 bootstrap replicates. The generated trees were edited with Figtree version 1.4.4.
